# Persimmon Fruit Powder May Substitute Indolbi, a Synthetic Growth Regulator, in Soybean Sprout Cultivation

**DOI:** 10.3390/molecules22091462

**Published:** 2017-09-03

**Authors:** Il-Doo Kim, Sanjeev Kumar Dhungana, Yong-Sung Park, Dong Joon Kim, Dong-Hyun Shin

**Affiliations:** 1 International Institute of Agricultural Research & Development, Kyungpook National University, Daegu 41566, Korea; ildookim@hanmail.net; 2 School of Applied Biosciences, Kyungpook National University, Daegu 41566, Korea; sanjeevdhungana@yahoo.com (S.K.D.); pjyjsj3@gmail.com (Y.-S. P.); 3 Department of Tourism Management, Yeungnam University College, Daegu 42415, Korea; djkim@ync.ac.kr

**Keywords:** Indolbi, isoflavone, nutritional value, persimmon fruit powder, soybean sprout, yield

## Abstract

Soybean sprouts are a major food item in Korea. Various studies have been carried out to enhance their yield and nutritional values. The objective of the present study was to examine the influence of persimmon fruit powder and Indolbi, a synthetic plant growth regulator, on the yield and nutritional value of soybean sprouts. Seeds were soaked in tap water containing 0.5%, 1.0%, 2.5% and 5.0% (*w*/*v*) persimmon fruit powder and the samples were named as PT-1, PT-2, PT-3, and PT-4, respectively. The yield increment was almost doubled in PT-3 and PT-4 than in the Indolbi treated sprouts on basis of the control. Vitamin C, isoflavones, and total phenolic contents as well as antioxidant potentials (determined by 1,1-diphenyl-2-picrylhydrazyl and superoxide anion radical scavenging assays) were also significantly (*p* < 0.05) higher in PT-3 compared to the Indolbi treatment and the control. However, total free amino acid and magnesium contents of Indolbi- applied sprouts were higher than in the fruit powder treatments. The overall results of the present study showed that persimmon fruit powder can be an option to enhance the yield and nutritional value of soybean sprouts since, due to potential health hazards, the use of synthetic chemicals like Indolbi is less preferred than the natural products.

## 1. Introduction

Soybean (*Glycine max* L.) sprouts have been established as one of the major vegetables in Korea for a long time. Therefore, large amounts of soybean sprouts are produced in Korea each year [1]. Various unwanted constituents or their functions in soybean seeds, for instance, trypsin, chymotrypsin, lipoxygenase activity, phytic acid, and oligosaccharides can be removed or reduced during germination [2,3,4]. On the other hand, the amounts of other desirable constituents such as vitamins, phytosterols, tocopherols, and isoflavones could be increased during the period [3,5]. In addition to nutritional value, germination also improves textural and organoleptic characteristics of legume seeds [6,7,8,9].

Isoflavones are a vital group of phytochemicals in soybean sprouts. Consumption of soy products containing isoflavones reduces depressive symptoms during pregnancy [10]. High dietary isoflavone intake may significantly increase fertility in humans [11]. Intake of soy foods, high in isoflavone/polyphenolic molecules, from conception was found to be advantageous in reducing prostate weight and white adipose tissue deposition while increasing testicular weight, Sertoli cell area, and seminiferous tubule volume in rats [12]. In another report, isoflavones from soy foods were found to be associated with reduced risk of ovarian cancer in southern Chinese women [13].

Soybean sprouts can be produced without using advanced technologies and also can be grown in a considerably short time which are added advantages in their production. Soybean sprouts are an inexpensive food sources to supply dietary functional materials [14]. Soybean sprouts can also be used in producing cosmetic products with anti-aging and skin whitening effects [15].

Various experiments have been conducted to enhance the nutritional and functional properties of soybean sprouts since germination is one of the economical and effective methods to increase its food value [16]. Calcium chloride was used to treat seeds and sprouts to enhance the yield and quality characteristics of soybean sprouts [17]. Soybean seeds were exposed to ultrasound treatment and this improved the edibility and nutritional quality of sprouts [18]. Macronutrients, phytochemicals, and antioxidant activities of soybean sprouts were manipulated with or without light exposure [19]. Similarly, soybean seeds and sprouts were exposed to gamma radiation to increase the microbial safety [20]; zinc sulphate solution was sprayed to enrich the zinc content in soybean sprouts [21]; various light treatments were imposed to influence the isoflavone content in soybean sprouts [22]; bacterial strains were inoculated to investigate their effect on bioactive compounds and antioxidant potentials of soybean sprouts [23]; grapefruit seed extract, chitosan, and phosphate buffer treatments increased the yield and inhibited sprout rot [24]; quality of soybean sprouts was increased with *ginseng* treatment [25]. In addition, various synthetic plant growth regulators such as Indolbi (INB) and 6-benzylaminopurine are also used to enhance yield and nutritional values of soybean sprouts [26]. However, due to potential health hazards, the use of such synthetic chemicals in food products is generally less preferred by consumers.

Very limited studies have been conducted to investigate the effect of persimmon fruit on soybean sprouts although persimmon is rich in different nutrients and phytochemicals [27,28,29,30]. Persimmon also contains a flavonoid fisetin, an anticancer agent [31]. Our recent report [32] shows that seed treatment with persimmon fruit powder enhances the yield and food values of soybean sprouts. In addition to the yields, nutrients, and antioxidant potentials considered in the previous study [32], the present study compares the color values and isoflavone content of soybean sprouts produced by treating seeds with persimmon fruit powder and that of INB-treated sprouts.

## 2. Results and Discussion

### 2.1. Yield and Moisture and Vitamin C Contents

The effects of seed treatment with persimmon fruit powder and INB on the yield, moisture and vitamin C contents of soybean sprouts are shown in Table 1. The sprout samples named as PT-1, PT-2, PT-3, and PT-4 were produced from the seeds soaked in tap water containing 0.5%, 1.0%, 2.5% or 5.0% (*w*/*v*) persimmon fruit powder, respectively. The fruit powder and INB treatment significantly (*p* < 0.05) increased the yield and vitamin C content after 6 d of germination. However, the effect of the treatments was non-significant (*p* > 0.05) for moisture content in the sprouts. The yield and vitamin C content were significantly higher in PT-3 than in INB-treated sprouts. The yield increment was almost doubled in PT-3 (115.9%) compared to INB (108.5%)-treated sprouts based on the yield of control (100%).

The amount of vitamin C content of soybean sprouts was significantly higher in PT-3 (18.11 mg/100 g) than in INB (16.76 mg/100 g). The high yield and vitamin C content attributed to the persimmon powder treatment might be due to some growth-enhancing substances present in the persimmon fruits. The results of this experiment show that higher yield with enhanced vitamin C in soybean sprouts could be produced using persimmon fruit powder compared to Indolbi, a synthetic plant growth regulator.

### 2.2. Color Value of Soybean Sprouts

In Hunter’s color value measurement, the ‘L’ is the measure of lightness, from completely opaque (0) to transparent (100); the ‘a’, measure of redness (‘−a’ greenness); and the ‘b’, measure of yellowness (‘−b’ blueness). Lightness value of INB or the fruit powder-treated soybean sprouts was significantly (*p* < 0.05) low as compared to the control. The redness value of soybean sprouts was not significantly (*p* > 0.05) affected by the INB treatment, however was significantly (*p* < 0.05) low for fruit powder-treated sprouts compared to the control. The yellowness value of the control was significantly (*p* < 0.05) high compared to the other treatments. However, the yellowness value of PT-3 was significantly (*p* < 0.05) high compared to INB (Table 2). The effect of persimmon or INB treatments on color development of soybean sprouts, in the present study, was not well known. Persimmon fruit powder enhanced the yellowness of sprouts which is one of the preferred traits in soybean sprouts.

### 2.3. Free Amino Acid Composition

A total of 37 free amino acids were detected, out of which eight, eight, and 21 were essential, non-essential, other free amino acids, respectively (Table 3). The amount of individual amino acid varied significantly among the treatments. The highest amount of total free amino acids was found in INB (579.99 mg/g) followed by PT-3 (533.75 mg/g) and the control (505.86 mg/g). Similarly, the ratio of essential to non-essential amino acids was the highest for INB (0.37) followed by PT-3 (0.36). The ratio for the control was 0.34. Foods with high ratios of essential to non-essential amino acids are considered well balanced for protein deposition [33]. The most abundant amino acid in the sprout samples was glutamic acid. γ-Amino-*n*-butyric acid (GABA) is primarily synthesized in plant tissues by decarboxylation of glutamic acid in the presence of glutamate decarboxylase [34].

INB significantly (*p* < 0.05) increased both the glutamic acid and GABA contents compared to other treatments and the control. GABA and glycine, other amino acids which were also increased with INB and -PT-3 although not significantly, are related to learning and memory, stroke and neurodegenerative diseases; relieving anxiety, sedation, anticonvulsant, and muscle relaxation functions [35,36,37]. GABA rich foods are also considered as brain foods and possess bioactive functions such as regulating blood cholesterol and suppressing blood pressure, improving cerebral blood flow, reducing insomnia, depression, and pain [38]. GABA is also reported to have anti-diabetic effect [34]. Amount of amino acid is one of the major components determining the nutritional potentials of fruits, vegetables, and foods [39]. The results of this study showed that amino acid content in soybean sprouts can be improved with INB and persimmon fruit powder. However, the specific mechanisms by which the free amino acid profile were influenced in the soybean sprouts were not understood.

### 2.4. Mineral Content

The amount of minerals detected in the soybean sprouts are shown in Table 4. The most abundant mineral in the sprout samples was K (15553.43–21127.18 mg/kg) followed by Ca (2110.90‒3165.31 mg/kg). The values in the brackets are the minimum and maximum for each mineral considered. INB and persimmon fruit powder significantly (*p* < 0.05) reduced the amount of Ca (except in PT-4), Cu, and K in the sprouts. The reduction in these three minerals might be due to increment in the amount of other minerals (Table 4). Although the amount of total mineral content in PT-1 was higher than that in INB, total mineral content for the treatments was less than the control. However, amount of minerals such as Zn and Fe, which are often lacking in the human diet [40] has been increased with fruit powder treatment (PT-1). The reason for the reduced mineral content with INB and persimmon fruit powder was not well understood.

### 2.5. Isoflavone Content

Effect of INB and persimmon fruit powder on the amount of isoflavones has been shown in Table 5. Daidzin (331.6–386.7 mg/kg) was the most abundant isoflavone, followed by genistin (260.2–316.4 mg/kg) and glycitin (83.0–95.0 mg/kg) in the soybean sprouts. The values in the brackets are the minimum and maximum for each isoflavone considered. INB did not influence the amount of isoflavones significantly, however, persimmon fruit powder significantly (*p* < 0.05) increased the total amount compared to the control. Moreover, the amount of genistin was significantly lower in INB (260.2 mg/kg) than in the control (273.1 mg/kg).

Soy isoflavones possess the potentiality of preventing aging, cardiovascular diseases, and cancer since they are capable of scavenging free radicals from the human body [41]. Isoflavones are also beneficial because of their potential roles against chronic diseases like osteoporosis and hypercholesterolemia as well as alleviation of postmenopausal syndromes [42,43,44]. Use of persimmon fruit powder at varied concentrations, in the present study, has consistently increased the total isoflavones content. Results of the present study reveal that persimmon fruit powder can effectively be used to enhance the isoflavones content in soybean sprouts.

### 2.6. DPPH and Superoxide Anion Radical Scavenging Activities and Total Phenolic Content

Free radical scavenging potentials of soybean sprouts were measured through DPPH and superoxide anion. The free radical scavenging potentials and total phenolic content of INB and persimmon fruit-treated soybean sprouts were significantly (*p* < 0.05) different (Table 6). The DPPH free radical scavenging potential of the fruit powder treated sprouts (PT-3 and PT-4) was significantly (*p* < 0.05) higher than that of INB-treated and the control samples. Similarly, the superoxide anion scavenging potential was significantly increased in PT-1 (13.94%), PT-3 (15.77%), and PT-4 (20.04%) compared to the INB (11.06%) and the control (9.13%). Total phenolic content of PT-3 (355.46 µg GAE/g) was significantly higher than that of INB (343.48 µg GAE/g) and the control (326.20 µg GAE/g). Reactive oxygen species (ROS) such as hydrogen peroxide, hydroxyl radical, and singlet oxygen, basically produced from superoxide anions, induce oxidative damage in lipids, proteins, and DNA. The elevated levels of ROS can pose a risk to cells by lipids peroxidation, proteins oxidation, nucleic acids destruction, enzyme inhibition, programmed cell death activation pathway, and eventually cells death [45,46]. The high phenolic content in PT-3 might be due to high amount of phenols present in the persimmon fruits [47,48] as in zinc sulphate treatment enhanced the zinc content in soybean sprouts [21]. Phenolic compounds possess antioxidant potentials in vegetables and other foods [49,50]. Therefore, the total phenol content may have contributed to higher DPPH and superoxide anion radical scavenging potentials of persimmon fruit treated soybean sprouts.

## 3. Materials and Methods 

### 3.1. Experiment Materials and Reagents

Soybean (*Glycine max* L.) seeds of cultivar ‘Sowonkong’ were purchased from a local store in Deagu, Korea. The cultivar was released as a sprout cultivar in 1999 [51]. The mean seed weight was 12 g of 100 seeds. Persimmon fruits of cultivar ‘Sangjudungsi’ were obtained from Sangju Persimmon Experiment Station (Gyeongsangbukdo Agricultural Research and Extention Services, Sangju, Korea). The freeze-dried fruits were ground into powder using a commercial grinder (Speed Rotor Mill, Model KT-02A, Seishin, Fukuoka, Japan) and strained through a 100-mesh sieve. The following chemicals and reagents were obtained for the present study: Indolbi (INB) (Sammi Chemical Industries, Incheon, Korea); 1,1-diphenyl-2-picrylhydrazyl (DPPH), Folin-Ciocalteau-reagent, isoflavone standards (≥95% purity, Sigma-Aldrich Corporation, St. Louis, MO, USA), dimethyl sulfoxide (DMSO), and pyrogallol (Sigma-Aldrich Corporation, St. Louis, MO, USA); amino acid standards (Wako Pure Chemical Industries, Ltd., Osaka, Japan). All the other chemicals were of analytical grade.

### 3.2. Cultivation of Soybean Sprouts

Sprouts were grown following the method described by Kim et al. [32] with some modifications. Soybean seeds were cleaned from any debris or external materials. One kilogram of intact seeds (for each treatment and replication) were carefully washed with tap water separately for surface cleaning. The seeds were steeped in tap water containing different amounts of persimmon fruit powder or tap water alone for 8 h. INB was diluted in tap water at the ratio of 1:120 (*v*/*v*) for spraying the germinating seeds. The samples were named as control (seeds soaked in tap water alone), INB (seeds soaked in tap water for 8 h and thoroughly sprayed with 160 mL of diluted INB per kilogram of seed after 24 h), PT-1 (seeds soaked in water containing 0.5% (*w*/*v*) persimmon powder), PT-2 (seeds soaked in water containing 1.0% (*w*/*v*) persimmon powder), PT-3 (seeds soaked in water containing 2.5% (*w*/*v*) persimmon powder), and PT-4 (seeds soaked in water containing 5.0% (*w*/*v*) persimmon powder). After soaking, the seeds were kept in 15-L plastic buckets with a perforated base for the sprout cultivation. The seeds and sprouts were watered with two hoses of 1-cm diameter for 2 min every 3 h. Soybean sprouts were grown at room temperature 22 ± 2 °C for 6 d. Sprout sample powders were prepared for physicochemical studies. The fresh sprouts were kept at −70 °C and subjected to freeze drying. The freeze-dried sprouts were powdered using a commercial grinder (HIL-G-501, Hanil Co., Seoul, Korea) and strained using a 100-mesh sieve. The samples were kept into airtight sample bottles and stored at −20 °C until analyses.

### 3.3. Measurement of Sprout Yield 

The fresh yield of soybean sprouts was measured by deducting the weight of the empty bucket from the weight of each bucket containing sprouts. Yield was measured after 6 d.

### 3.4. Determination of Moisture and Vitamin C Content

Moisture content of soybean sprouts was determined following the method of AOAC [52] with some modifications. Fresh sprouts (5.0 g) was oven dried until constant weight. After drying, the moisture content was calculated using the following formula:

(1) $$\mathrm{Moisture}\;\left(\%\right)=\left[\frac{Wb-Wa}{Wb}\right]\times 100,$$

where, *Wb* = weight (g) of sprout before drying and *Wa* = weight (g) of sprout after drying.

Vitamin C content was determined following the standard method [53] and reported as mg/100 g fresh weight. Five grams of sample powder was blended in 7.5 mL of 3% metaphosphoric acid solution and homogenized (AM-8, Nihonseike Kaisha, Tokyo, Japan). The homogenate was extracted in 12.5 mL after filtration. Six milliliters of the extract was then titrated with 0.025% of 2,6-dichloroindophenol. In this reaction, vitamin C in the extract was oxidized and the indophenol dye reduced to a colorless compound.

### 3.5. Color Measurement

L* (lightness), a* (redness, + or greenness, −), and b* (yellowness, + or blueness, −) values of sample powders were measured using a Chroma meter (CR-300, Minolta Corp., Osaka, Japan). A Minolta calibration plate (YCIE = 94.5, XCIE = 0.3160, YCIE = 0.330) and a HunterLab standard plate (L* = 97.51, a* = −0.18, b* = +1.67) were used to standardize the instrument using a D65 illuminant [54]. Color values were measured on 3 zones of powder sample and mean values were calculated.

### 3.6. Determination of Free Amino Acid Content

Free amino acids were analyzed following the procedure of Je et al. [55] with some modifications. One gram of sprout sample was hydrolyzed with 6 N HCl (10 mL) in a sealed-vacuum ampoule at 110 °C for 24 h. The HCl was removed from the hydrolyzed sample on a rotary evaporator, the content was mixed with 0.2 M sodium citrate buffer (pH 2.2) to make a volume of 5.0 mL. The mixture was passed through a C-18 Sep Pak (Waters Co., Milford, MA, USA) cartridge and filtered through a 0.22 μm membrane filter (Millipore, Billerica, MA, USA). Amino acids were determined using an automatic amino acid analyzer (Biochrom-20, Pharmacia Biotech Co., Uppsala, Sweden).

### 3.7. Determination of Mineral Content

Mineral content was determined following the method of Skujins [56] with some modifications. Sample powder (0.5 g) and HNO_3_ (15.0 mL) were mixed into a cup. The mixture was diluted with equal volume of distilled water. Mineral concentrations were determined using inductively coupled plasma atomic emission spectrometer (ICP AES: Varian Vista, Varian Australia, Victoria, Australia).

### 3.8. Determination of Isoflavone Content

Isoflavones were measured using High Performance Liquid Chromatography (HPLC) following the procedure described by Jiao et al. [57]. Sample powder (0.2 g) was extracted with 6.0 mL of 80% methanol by ultrasonic-assisted method at 40 °C for 30 min and centrifuged. The supernatant was filtered through a 0.45 µm membrane filter (Millipore) before HPLC analysis. The isoflavones were analyzed under the following conditions of HPLC: flow rate 1 mL/min; the mobile phase: solvent A —aqueous acetic acid (0.1%), and solvent B—acetic acid in acetonitrile (0.1%). HPLC running condition consisted of a gradient of 13–35% B during a 52 min period; oven temperature was 35 °C. The injection volume was 20 µL. The eluted isoflavones were detected at 260 nm. Each peak was identified by the retention time and the characteristic UV spectrum in comparison with the corresponding standards.

### 3.9. Determination of DPPH Free Radical Scavenging Potential

The DPPH free radical scavenging activity of sample was measured following the method given by Blois [58] with some modifications. One gram of sample was extracted in 10 mL of absolute methanol in a shaking incubator (150 rpm, 25 °C) for 8 h. The mixture was centrifuged (3000 rpm, 15 min), and the supernatant was filtered through a 0.2-µm syringe filter (Waters Co.). Equal volumes of sample extract (0.1 mL) and freshly prepared 0.1% (*w*/*v*) of methanolic solution of DPPH (0.1 mL) were put into microplate wells and incubated at room temperature in the dark for 30 min. The absorbance was measured at 517 nm using a microplate spectrophotometer (Multiskan GO, Thermo Fisher Scientific, Vantaa, Finland). Equal proportions of methanol and sample extract as well as methanol and DPPH were mixed to measure the absorbance of control and blank, respectively. The DPPH radical-scavenging activity was calculated from the absorbance using the following equation:

Scavenging activity (%) = [1 − (*A* − *A_b_*)/*A_o_*] × 100, (2)

where, *A_o_* is the absorbance of methanol and DPPH without sample (blank), *A* is the absorbance of sample extract and DPPH, and *A_b_* is the absorbance of sample extract and methanol (control).

### 3.10. Determination of Superoxide Anion Scavenging Activity

Measurement of superoxide anion scavenging activity was based on the method described by Li [59]. A sample extract (0.3 mL) and 2.61 mL of 50 mM phosphate buffer (pH 8.24) were added into freshly prepared 90 μL of 3 mM pyrogallol prepared in 10 mM HCl solution. The inhibition rate of pyrogallol auto-oxidation was measured at 325 nm (UV-1700, Shimadzu, Tokyo, Japan). Absorbance of each extract was recorded at every 1 min interval for 10 min and the increment of absorbance was calculated by the difference.

### 3.11. Determination of Total Phenolic Content

Total phenols in the sample powders were determined following the Folin-Ciocalteau method [60]. The sample extract was prepared as in the DPPH analysis. Fifty microliters of sample extract was added to 250 μL of 1 N Folin-Ciocalteau reagent. After 1 min, 750 μL of 20% (*w*/*v*) aqueous Na_2_CO_3_ was added, and the volume was made to 5.0 mL with distilled water. After 2 h of incubation at 25 °C in dark, the absorbance was measured at 760 nm (Multiskan GO, Thermo Fisher Scientific, Vantaa, Finland). Total phenols were determined as gallic acid equivalent (μg GAE /g dry sample).

### 3.12. Statistical Analysis 

Data were subjected to analysis of variance (ANOVA) using SAS 9.3 (SAS Institute, Cary, NC, USA) and significant differences between means at 5% probability were analyzed using Tukey test. Average values of triplicate measurements were considered for statistical analysis unless otherwise mentioned.

## 4. Conclusions

This study investigated the effects of Indolbi and persimmon fruit powder on the yield and nutritional values of soybean sprouts. The results of the present study showed that soaking soybean seeds in water containing persimmon fruit powder or applying Indolbi can significantly increase the yield, vitamin C, free amino acids, minerals, isoflavones, and total phenolic contents of soybean sprouts. Based on the yield and nutritional parameters investigated in the present study, the effect of persimmon fruit powder, especially at 2.5% (*w*/*v*) in the water, was superior compared to that of Indolbi. Moreover, because of potential health hazards, the use of synthetic chemicals like Indolbi is less preferred than that of natural products by consumers, therefore, persimmon fruit powder could be a good option to enhance the yield and nutritional value of soybean sprouts.

## Figures and Tables

**Table 1:** Effect of Indolbi (INB) and persimmon fruit powder on yield and moisture and vitamin C contents of soybean sprouts cultivated for 6 days.

Sample ^1^	Total Weight (g)	Moisture (%)	Vitamin C (mg/100 g Fresh Weight)
Control	5523 ± 51d ^2^ (100.0%)	87.21 ± 0.04a	16.11 ± 0.21c
INB	5995 ± 52b (108.5%)	87.00 ± 0.18a	16.76 ± 0.52b
PT-1	5900 ± 30c (106.8%)	86.88 ± 1.05a	16.52 ± 0.39bc
PT-2	6140 ± 38b (111.1%)	87.11 ± 1.11a	16.99 ± 0.31b
PT-3	6300 ± 61a (114.0%)	86.99 ± 1.00a	18.11 ± 0.62a
PT-4	6402 ± 50a (115.9%)	87.12 ± 0.09a	17.29 ± 0.32ab

^1^ Control, soybean seeds soaked in tap water for 8 h; INB, seeds soaked in tap water for 8 h and thoroughly sprayed with 160 mL of diluted INB per kilogram of seed after 24 h; PT-1, soybean seeds soaked in tap water containing 0.5% (*w*/*v*) persimmon fruit powder for 8 h; PT-2, soybean seeds soaked in tap water containing 1.0% (*w*/*v*) persimmon fruit powder for 8 h; PT-3, soybean seeds soaked in tap water containing 2.5% (*w*/*v*) persimmon fruit powder for 8 h; PT-4, soybean seeds soaked in tap water containing 5.0% persimmon fruit powder for 8 h. Percentage, for total weight, in parentheses denotes the variation in sprout yields in respect to the Control. ^2^ Values are expressed as mean ± standard deviation of three replicates. Values followed by different letters (a, b, c, and d) in the same column indicate significant difference (*p* < 0.05, ANOVA, Tukey test).

**Table 2:** Hunter’s color values of soybean sprouts cultivated with Indolbi (INB) or persimmon fruit powder treatment.

Sample ^1^	Color Value ^2^		
	L*	a*	b*
Control	62.05 ± 0.05a ^3^	−1.42 ± 0.07a	20.82 ± 0.03a
INB	59.13 ± 0.06b	−1.37 ± 0.03a	19.52 ± 0.21c
PT-1	59.18 ± 0.13b	−2.34 ± 0.04e	18.95 ± 0.05e
PT-2	59.42 ± 0.22b	−1.98 ± 0.02c	19.19 ± 0.03d
PT-3	58.66 ± 0.81b	−1.60 ± 0.01b	20.05 ± 0.04b
PT-4	59.45 ± 0.71b	−2.03 ± 0.04d	19.53 ± 0.06c

^1^ Samples are defined in Table 1. ^2^ L*, lightness (100, white; 0, black); a*, redness (−, green; +, red); b*, yellowness (−, blue; +, yellow). ^3^ Values are expressed as mean ± standard deviation ofthree replicates. Values followed by different letters (a, b, c, d, and e) in the same column are significantly different (*p* < 0.05, ANOVA, Tukey test).

**Table 3:** Free amino acid composition (mg/g of dry weight) of soybean sprouts cultivated with Indolbi (INB) or persimmon fruit powder treatment.

Amino Acid		Sample ^1^				
	Control	INB	PT-1	PT-2	PT-3	PT-4
**Essential Amino Acid**						
l-Histidine	22.98 ± 2.0ab	26.26 ± 1.7a	18.20 ± 1.8c	21.95 ± 0.8b	24.98 ± 2.9a	20.68 ± 0.8bc
l-Isoleucine	15.20 ± 0.0c	18.59 ± 0.8a	9.89 ± 1.12f	13.31 ± 0.7d	16.62 ± 0.3b	12.11 ± 0.1e
l-Leucine	10.70 ± 0.1b ^2^	13.03 ± 0.2a	6.72 ± 0.2e	8.86 ± 0.1c	11.52 ± 1.0b	7.67 ± 0.4d
l-Lysine	8.09 ± 0.3b	9.67 ± 0.4a	6.63 ± 0.5bc	7.24 ± 0.6b	7.93 ± 0.4b	6.29 ± 0.2c
l-Methionine	2.31 ± 0.0c	2.69 ± 0.0a	1.99 ± 0.0c	2.08 ± 0.0d	2.44 ± 0.0b	1.93 ± 0.0f
l-Phenylalanine	22.83 ± 1.4b	30.18 ± 2.1a	11.15 ± 1.0d	17.91 ± 0.3c	23.50 ± 1.6b	15.69 ± 2.0c
l-Threonine	12.57 ± 1.0a	12.96 ± 0.9a	13.44 ± 1.3a	11.36 ± 2.0a	13.59 ± 1.0a	10.18 ± 1.2b
l-Valine	21.62 ± 1.3c	26.60 ± 1.8a	13.74 ± 1.8c	19.06 ± 1.0c	23.96 ± 0.5b	16.99 ± 1.0d
**Sub-total**	**116.30**	**139.98**	**81.76**	**101.77**	**124.54**	**91.54**
**Non-essential Amino Acid**						
Glycine	2.97 ± 0.3ab	3.68 ± 0.4a	2.54 ± 0.5b	2.91 ± 0.2b	3.29 ± 0.4a	1.99 ± 0.4c
l-Alanine	28.63 ± 2.1a	30.09 ± 2.1a	19.85 ± 0.9c	24.29 ± 1.3b	29.04 ± 1.0a	19.35 ± 0.9c
l-Arginine	51.08 ± 1.9b	62.85 ± 2.0a	37.37 ± 1.9d	43.67 ± 1.7c	49.52 ± 2.8b	43.65 ± 1.6c
l-Aspartic acid	25.15 ± 1.0a	27.13 ± 1.0a	20.69 ± 0.8c	23.74 ± 0.9b	26.93 ± 0.8a	25.84 ± 2.1ab
l-Glutamic acid	200.61 ± 5.1b	221.39 ± 6.0a	144.06 ± 3.7d	165.49 ± 5.2c	216.39 ± 4.2b	157.59 ± 3.7c
l-Serine	20.37 ± 1.0a	21.85 ± 1.1a	15.81 ± 1.0c	19.14 ± 0.9a	21.86 ± 1.3a	18.15 ± 0.9b
l-Tyrosine	2.79 ± 0.1b	3.39 ± 0.3a	2.54 ± 0.3b	2.59 ± 0.3b	2.99 ± 0.5ab	2.18 ± 0.1c
Proline	7.32 ± 0.9b	8.59 ± 0.3a	5.12 ± 0.2d	5.90 ± 0.4c	0.66 ± 0.9b	5.41 ± 3.4cd
**Sub-total**	**338.92**	**379.97**	**248.98**	**287.73**	**350.68**	**274.16**
**Other Free Amino Acid**						
1-Methyl-l-histidine	1.29 ± 0.1b	1.81 ± 0.1a	0.90 ± 0.0d	1.11 ± 0.0c	1.32 ± 0.2b	0.88 ± 0.2d
Aminoisobutyric acid	0.35 ± 0.0c	0.39 ± 0.1c	0.57 ± 0.0b	0.63 ± 0.2ab	0.70 ± 0.1a	0.58 ± 0.1ab
Ethanolamine	3.03 ± 0.0c	3.80 ± 0.1a	3.11 ± 0.3b	3.41 ± 0.2b	3.03 ± 0.3bc	3.21 ± 1.0ab
l-Anserine	3.46 ± 0.1b	3.78 ± 0.3b	2.78 ± 0.3c	3.60 ± 0.6b	4.51 ± 0.5a	2.62 ± 0.4c
l-Carnosine	ND ^3^	0.14 ± 0.1b	0.10 ± 0.0c	0.14 ± 0.0b	0.17 ± 0.0a	0.07 ± 0.0d
l-α-Aminoadipic acid	2.54 ± 0.0b	3.28 ± 0.1a	2.47 ± 0.1b	2.48 ± 0.2b	2.51 ± 0.3b	2.57 ± 0.1b
l-α-Amino-*n*-butyric acid	0.98 ± 0.0b	1.05 ± 0.1b	0.74 ± 0.0d	1.06 ± 0.0b	1.37 ± 0.0a	0.88 ± 0.0c
O-Phosphoethanolamine	0.51 ± 0.0c	0.32 ± 0.0e	0.41 ± 0.0d	0.52 ± 0.0c	0.61 ± 0.0b	0.72 ± 0.0a
Urea	22.01 ± 0.2a	22.81 ± 0.6a	14.83 ± 0.5d	18.47 ± 0.3b	21.53 ± 0.7a	16.85 ± 0.4c
β-Alanine	2.38 ± 0.1b	2.56 ± 0.2a	1.71 ± 0.5c	2.08 ± 0.3ab	2.49 ± 0.3ab	1.63 ± 0.3c
γ-Amino-*n*-butyric acid	14.09 ± 1.3bc	21.10 ± 1.0a	12.56 ± 1.0c	12.93 ± 0.7c	14.27 ± 1.0bc	15.71 ± 1.0b
**Sub-total**	**50.64**	**61.04**	**40.18**	**45.95**	**58.53**	**45.70**
**Total**	**505.86**	**579.99**	**369.92**	**435.45**	**533.75**	**411.40**

^1^ Samples are defined in Table 1. ^2^ Values are expressed as mean ± standard deviation of two replicates. Values followed by different letters (a, b, c, d, and e) in the same row are significantly different (*p* < 0.05, ANOVA, Tukey test). ^3^ Non detected.

**Table 4:** Mineral contents (mg/kg of dry weight) of soybean sprouts cultivated with Indolbi (INB) or persimmon fruit powder treatment.

Element	Sample ^1^					
	Control	INB	PT-1	PT-2	PT-3	PT-4
Ca	3021.90 ± 10.2a ^2^	2840.81 ± 12.0b	2110.90 ± 9.2d	2510.31 ± 15.1c	2831.41 ± 18.1b	3165.31 ± 8.1a
Cu	45.21 ± 0.4a	21.09 ± 0.1d	29.35 ± 0.1b	21.54 ± 0.0c	24.94 ± 0.1b	19.06 ± 0.0e
Fe	57.63 ± 0.94b	54.57 ± 1.07c	61.48 ± 0.51a	51.46 ± 0.30d	54.95 ± 0.73c	48.51 ± 0.04d
K	21127.18 ± 32.1a	18191.02 ± 82.1c	20591.88 ± 30.9b	16798.96 ± 85.7d	18069.51 ± 115.2c	15553.43 ± 163.2e
Mg	1202.32 ± 49.2b	1283.74 ± 10.6a	1218.83 ± 16.4b	1208.52 ± 18.6b	1220.60 ± 3.6b	1151.98 ± 5.3c
Mn	48.67 ± 0.8c	48.30 ± 1.1c	54.86 ± 0.5a	43.00 ± 0.3d	50.76 ± 0.7b	42.48 ± 0.6d
Na	560.35 ± 16.3c	422.84 ± 5.9e	668.67 ± 1.1a	476.27 ± 5.7d	581.64 ± 2.7b	407.40 ± 6.5f
Zn	64.01 ± 1.1b	55.04 ± 0.9d	68.42 ± 0.9a	54.74 ± 0.9d	62.39 ± 0.9c	47.69 ± 0.9e
**Total**	**26127.27**	**22917.41**	**24804.39**	**21164.80**	**22896.20**	**20435.86**

^1^ Samples are defined in Table 1. ^2^ Values are expressed as mean ± standard deviation of two replicates. Values followed by different letters (a, b, c, d, e, and f) in the same row are significantly different (*p* < 0.05, ANOVA, Tukey test).

**Table 5:** Isoflavone content of soybean sprouts cultivated with Indolbi (INB) or persimmon fruit powder treatment.

Sample ^1^	Isoflavone Content (mg/kg)						
	Daidzin	Daidzein	Genistin	Glycitin	Glycitein	Genistein	Total
Control	344.4 ± 9.3b ^2^	18.4 ± 5.4b	273.1 ± 3.6c	85.9 ± 6.1ab	10.5 ± 1.3a	34.7 ± 2.9a	767.0 ± 28.6b
INB	331.6 ± 6.5b	20.7 ± 6.9ab	260.2 ± 4.9d	83.0 ± 5.3b	11.0 ± 2.0a	36.1 ± 4.2a	742.6 ± 28.2b
PT-1	383.4 ± 5.9a	25.0 ± 3.1a	281.0 ± 5.0c	94.3 ± 7.0ab	9.6 ± 3.1a	23.4 ± 5.1bc	816.7 ± 29.0a
PT-2	370.1 ± 8.4a	22.3 ± 2.6ab	297.3 ± 8.6b	92.7 ± 6.2ab	9.7 ± 1.1a	24.3 ± 2.1bc	816.4 ± 28.7a
PT-3	373.9 ± 14.0a	18.7 ± 1.8b	311.7 ± 2.3a	86.7 ± 5.4ab	10.3 ± 1.0a	22.2 ± 1.9c	823.5 ± 26.6a
PT-4	386.7 ± 12.1a	21.6 ± 2.2ab	316.4 ± 6.0a	95.0 ± 6.7a	9.8 ± 2.1a	26.9 ± 1.8b	856.4 ± 30.5a

^1^ Samples are defined in Table 1. ^2^ Values are expressed as mean ± standard deviation of three replicates. Values followed by different letters (a, b, c, and d) in the same column are significantly different (*p* < 0.05, ANOVA, Tukey test).

**Table 6:** Scavenging activities of reactive oxygen species and total phenolic contents of soybean sprouts cultivated with Indolbi (INB) or persimmon fruit powder treatment.

Sample ^1^	% Inhibition ^2^		Total Phenol Content (µg GAE ^3^/g of Sample)
	DPPH	O_2_^-^	
Control	75.90 ± 1.2d ^4^	9.13 ± 0.6e	326.20 ± 3.1c
INB	78.18 ± 0.4c	11.06 ± 0.2d	343.48 ± 1.4b
PT-1	77.41 ± 0.8cd	13.94 ±0.3c	298.92 ± 2.1e
PT-2	79.88 ± 1.0bc	11.54 ± 0.1d	328.41 ± 2.3c
PT-3	83.19 ± 0.3a	15.77 ± 0.5b	355.46 ± 0.9a
PT-4	80.03 ± 0.4b	20.04 ± 0.1a	313.44 ± 0.5d

^1^ Samples are defined in Table 1. ^2^ O_2_^-^: DPPH: DPPH free radical scavenging activity, Superoxide anion scavenging activity. ^3^ GAE: gallic acid equivalent. ^4^ Values are expressed as mean ± standard deviation of three replicates. Values followed by different letters (a, b, c, d, and e) in the same column are significantly different (*p* < 0.05, ANOVA, Tukey test).
